# Method for Determining the Weight of Functional Objectives on Manufacturing System

**DOI:** 10.1155/2014/242368

**Published:** 2014-08-28

**Authors:** Qingshan Zhang, Wei Xu, Jiekun Zhang

**Affiliations:** School of Management, Shenyang University of Technology, Shenyang 110870, China

## Abstract

We propose a three-dimensional integrated weight determination to solve manufacturing system functional objectives, where consumers are weighted by triangular fuzzy numbers to determine the enterprises. The weights, subjective parts are determined by the expert scoring method, the objective parts are determined by the entropy method with the competitive advantage of determining. Based on the integration of three methods and comprehensive weight, we provide some suggestions for the manufacturing system. This paper provides the numerical example analysis to illustrate the feasibility of this method.

## 1. Introduction

Manufacturing is the basic activity of an enterprise's survival and development [1]. Manufacturing system relates to the process of converting the input into the output by using manufacturing resources and the manufacturing process. The manufacturing process is a series of organized manufacturing activities around a product or service. As a process of “input-conversion-output,” it puts in a certain resource, which will be value-added through a series and various forms of transformation, providing for the society in some form of output. The manufacturing resources refer to the conditions of supporting manufacturing in the internal enterprise [2]. It is mainly made up of people, goods, content, and technology, as a supporting system of the manufacturing process.

Manufacturing system has obvious economic and social benefits and this would come true mainly through its function objective. The requirements of the enterprise environment and user mainly reflect in high efficiency, low cost, high quality, short delivery time, personalization, and green environmental protection. There are mutual connections, restrictions, and even contradictories under these objectives [3]. The decision of function objectives is that the original traditional manufacturing system pays close attention to the single coordination and balance between low cost and high efficiency. This already cannot satisfy the needs of the enterprises competition.

Establishing a scientific and effective method for manufacturing system function objective to determine weights is the vital basis and prerequisite of making strategic decisions, product development strategy, and manufacturing system optimization. It is necessary methods of implementing a differentiation competitive strategy to get customers, improve the market competitiveness and make it into the high-end link of a value chain. This paper tries to establish a kind of three dimensional weight decision-making methods, which are used to meet the function objective decision problems in the design of the manufacturing system.

## 2. Literature Review

Heijltjes and van Witteloostuijn [4] carried out multidimensional evaluation for production system structure and complexity of the process, further deepened production system layout ideas, so that it improved efficiency of making system functional objectives decision. Grigoroudis and Siskos [5] presented a multiobjective optimization decision of product line and hybrid assembly systems about species diversity of contemporary products and production systems complexity to counterpoise variety and complexity. Levis and Papageorgiou [6] developed a cost estimation model, the redesign of the product and craft can support investment decision analysis with better cost and value index; Hon [7] researched the factors of the relationship between service quality of productive service and the relationship between various factors; they constructed a service quality model of tangible, reliability, corresponding, assurance and empathy five latent variable based on SERVPREF scale and used SEM to do analysis of degree of reliability and model fit. Wacker and Sheu [8] divided the manufacturing system into planning subsystem and execution subsystem; they also have collected data from 16 countries' 768 global manufacturing enterprises, and they have done empirical analysis to the manufacturing system functional objectives, drawn a conclusion that running system had a significant role in the company's competitiveness, and determined the weights of seven objectives that may speed the transportation, delivery rate, low cost, quality, production flexibility, product flexibility, and new product manufacturing capabilities. Youssef et al. [9] discussed manufacturing system simulation methods and modeling techniques, simulation system structure, and three aspects of simulation algorithm; they researched on the relevant agent model structure of each simulation level by agent modeling methods and techniques, providing the basis for the group decision-making. Dangayach and Deshmukh [10] proposed a reconfigurable manufacturing system scalability planning approach which can gradually expand system capacity by reconfiguring existing systems and using genetic algorithm optimization algorithm to determine the most economical way to reconfigure the existing manufacturing system objective weight. Menguc et al. [11] analyzed quantitative description of decision level fusion and dynamic fusion feature by collaborative manufacturing process of integration services and service needs and discussed collaborative service-oriented decision fusion with the positive feedback from the fusion of tissue morphology innovative features and dynamic decision fusion mechanism. Acquaah and Yasai-Ardekani [12] studied “TMS, TDS, TPS, and Scientific Total Quality Management” on the basis of promoting the TPS and made it the key to decisions. Kalogeraki et al. [13] used DEA efficiency evaluation method to evaluate the operational efficiency of the parallel structure production system, digging greater potential of the overall system performance improvement than traditional CCR model. Babakus et al. [14] introduced weighting methods of the self-reconfigurable KMS based on relations with fuzzy demands and estimated Function Objective Analysis. Vichare et al. [15] made European companies their research object to propose four kinds of objective decision-making direction of making: aftermarket service to provide (ASPs), customer support provided (CSPs), outsourcing partners (OPs), development partners (DPs), and gave them conditions of use and weight. Um et al. [16] researched flexible manufacturing systems, made a quantitative judgment on the “flexible” in FNS, gave the “flexible” stochastic dynamic programming quantitative evaluation model, and provided a reference weight to the choice of flexible manufacturing system determining the expected investment and technical means. Deng et al. [17] used CQN model to propose a hybrid genetic algorithm for optimal allocation of FMS and explained route planning process based on production and cost optimization. Ortega [18] designed a good production system to protect environment and internal fit (product and market) and raised six areas of production decisions. Salunke et al. [19] researched manufacturing from the perspective of business performance and gave decision and optimization of SOP manufacturing enterprise configuration capabilities under an uncertain environment. To improve the performance of production systems, and to help managers improve work efficiency and system evaluation, Agus and Hassan [20] developed tree manufacturing system functional objectives improvement strategies; Wang et al. [21] researched production systems from the output rate and buffering capacity links and used system dynamics model raising maintenance strategies for unexpected failure and processing requirements and the variability of the production cycle. Claver-Cortés et al. [22] raised an integrated architecture based on agents and services guide and did research on manufacturing system objectives implement from the perspective of information systems and technology. Wang and Koren [23] considered that the costs, service levels, lead time, and innovation are the key value elements in manufacturing system functionality objectives centered on the overall performance of manufacturing systems. Claver-Cortés et al. [22] have conducted a performance value analysis on GMS, gave a weight to the green attribute, and raised the fact that GMS is more competitive than non-GMS. Esbjerg et al. [24] considered the problem of manufacturing companies to meet individual customer needs in mass customization production systems, put the arrangements of production down to production assignment of products, quantified user needs and business output, and gave a multiattribute product model. Bolloju et al. [25] constructed multiobjective dynamic unit objective decision based on scatter search against the characteristics of market product that short life cycle, customer demand for personalized, shorten lead times, and so forth. Nouri and Hong [26] defined system's capacity of input elements and output service as manufacturing capacity from the perspective of management and did analysis of each subsystem by System Dynamics Model carefully; they changed the pattern that most scholars build an index system, decomposed function objective decision which has divided manufacturing capabilities system in time, quality, and profit three subsystems. Yang and Chen [27] proposed a fuzzy soft set-based approach to prioritizing technical attributes in quality function deployment. In the aspect of identified objective weight, there are many research methods which are mainly focused on (1) carrying on weight distribution to function objective decision based on the expertise; (2) carrying on subjective and objective weighting method to determine by customer demand preferences evolve rule; (3) using their own expectations, that is, business leaders subjective desires, to determine the weight. The level of the objective weights is not to determined based on the case manufacturing system itself which is in or combined with external opportunities and own resources conditions.

**Figure 1:**
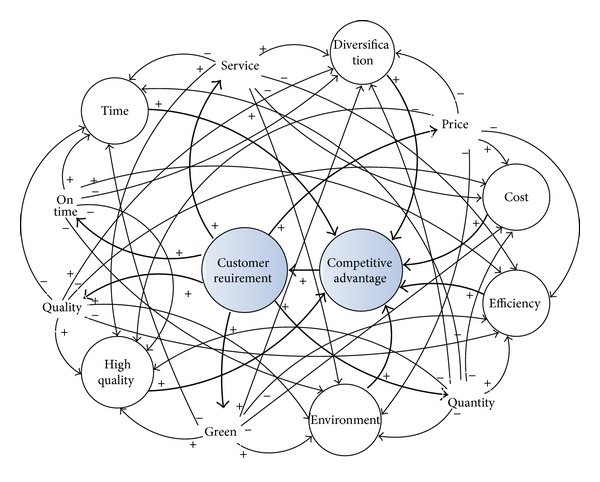
The function mechanism between the customer requirements and the system functional objectives.

In this paper, the influence of customer demand for manufacturing system function objective is combined with the strategic requirement of the enterprise manufacturing system, which solves the competitive advantage of manufacturing system and the intensity of competition for weight function objective, from the perspective of competitive strategy. Based on subjective fuzzy mathematics and objective entropy weight method, approach to decision-making was formed which is suitable for a dynamic environment. There is a link between customer demands and the interaction with the system functional objectives. Some demands and improving the system functional objectives showed a positive correlation, and some of the demands are no correlation between the objective system, and even some of them for the development of the system have a hindering effect for designed the system functional objectives. Using the dynamic principle of the system, we can clearly research the customer demand for intuitive system objectives and effects of competitive advantage, as shown in Figure 1.

## 3. Basic Principle

**Figure 2:**
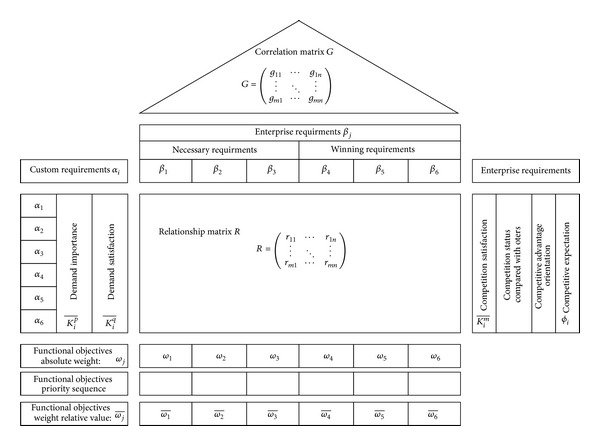
Quality house of module construction.

Through put the value function into prioritization to express the competitive advantage of enterprise, through the various functions gives different weights to realize, it is concluded that the enterprise itself under integrated balance function objective focusing on the direction and intensity, it is crucial for its survival and development. In response to this situation, the enterprise uses quality function deployment (quality function deployment, QFD) to design and manufacture, meet, or exceed customer expectations of products and manufacturing system function goal of the three factors as input part, through the principle of QFD system decision-making weight of each objective by function objective priority sequence. The main information contained in this house of quality is customer demand, competitive demand module, and enterprise demand modules, as shown in Figure 2.

Weights are determined by two aspects, on one hand as the elementary weight, while on the other hand as the directional weight. Consider

(1) $$\begin{array}{c} W^{T}=W^{A}+W^{S}, \end{array}$$

where  *W* ^*T*^ is the weight of manufacturing system functional orientation, *W* ^*A*^ is the basic weight, determined by customer needs and expectations of enterprises decision, and *W* ^*S*^ is the directional weight, determined by the enterprise strategic orientation.

Customers need determining the weight

(2) $$\begin{array}{c} W^{D}=\left[a_{1},a_{2},\ldots ,a_{m}\right]. \end{array}$$

Enterprises need determining the weigh

(3) $$\begin{array}{c} W^{C}={\left[b_{1},b_{2},\ldots ,b_{m}\right]}^{T}. \end{array}$$

The enterprise competitive advantage determines the weight

(4) $$\begin{array}{c} W^{S}=\left[c_{1},c_{2},\ldots ,c_{m}\right]. \end{array}$$

Calculated results of basic principle are

(5) $$\begin{array}{c} W^{A}=\frac{W^{D}\times W^{C}}{||W^{D}\times W^{C}||}=\frac{\left[a_{1}\times b_{1},a_{2}\times b_{2},\ldots ,a_{m}\times b_{m}\right]}{\sqrt{\sum_{i=1}^{m}a_{i}\times b_{i}}}. \end{array}$$

Therefore,

(6) WT=a1×b1∑i=1mai×bi+c1,a2×b2∑i=1mai×bi+c2,…, am×bm∑i=1mai×bi+cm.

As a consequence,

(7) $$\begin{array}{c} W_{i}^{T}=\frac{a_{i}\times b_{i}/\sqrt{\sum_{i=1}^{m}a_{i}\times b_{i}}+c_{i}}{\sqrt{\sum_{i=1}^{m}\left(a_{i}\times b_{i}/\sqrt{\sum_{i=1}^{m}a_{i}\times b_{i}}\right)+c_{i}}}. \end{array}$$

## 4. Model Building

### 4.1. Decomposition of Basic Weight

#### 4.1.1. Customer Weight

**Figure 3:**
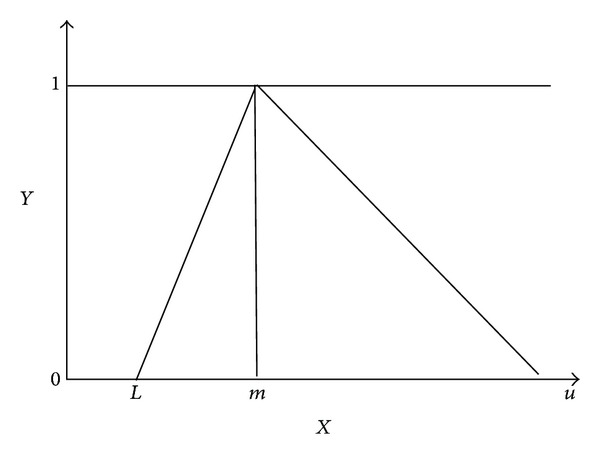
Triangular fuzzy numbers.

*(1) Triangular Fuzzy Numbers*. Membership function graph is as shown in Figure 1; fuzzy numbers α = (*l*, *m*, *u*) are called triangular fuzzy number, where *l*, *m*, *u* is a real number; 0 ≤ *l* ≤ *m* ≤ *u*, *m* is called the main value of triangular fuzzy number; *α*, *l*, and *u* are called the upper and lower bounds of *α*, (*m* − *l*); and (*u* − *m*) are called lower and upper limits of *α*. When *l* = *m* = *u*, α turns into the real number in the ordinary sense. When the value of (*u* − *l*) is larger, the triangular fuzzy number *α* = (*l*, *m*, *u*) is more blurred. *L* in this paper represents the clients' minimum expectations to the objective, *m* represents clients' most expectations, and *u* represents the customers' highest expectations to the objective, as in Figure 3.

*(2) Fuzzy Mean Value and Variance*. Fuzzy number has two kinds of distribution, uniform distribution and proportional distribution. We can define the respective fuzzy mean *m*(*α*) and variance *σ*(*α*) as follows.

 ① Uniform distribution is
  (8) mV(a)=∫S(a)xμa(x)dx∫S(a)μa(x)dx,σV2(a)=∫S(a)x2μa(x)dx∫S(a)μa(x)dx−mV2(a).
 ② Proportional distribution is
  (9) mP(a)=∫S(a)xμa(x)dx∫S(a)μa(x)dx,σP2(a)=∫S(a)x2μa(x)dx∫S(a)μa(x)dx−mP2(a).

*(3) Value Range*. In the value evaluation of each program, taking the ambiguity of the indicators and subjectivity into account, we use semantic judgment that is divided into seven specific grading criteria, namely, “very low” (VL), “low” (L), the “lower” (ML), “general” (M), “high” (MH), “high” (H), and the “very high” (VH); these seven variable semantic described as triangular fuzzy numbers, as illustrated in Table 1.

*(4) Clearance of Triangular Fuzzy Number*. We can compare the size of triangular fuzzy numbers by comprehensively utilizing the triangular fuzzy numbers mean, variance, and fuzzy limit coefficient composed with fuzzy information content:

(10) $$\begin{array}{c} E\left(a\right)=2\times \left(\mu_{a}^{2}\left(x_{0}\right)+\mu_{a^{\prime }}^{2}\left(x_{0}\right)\right)-1. \end{array}$$

Fuzzy information content is defined as: for the arbitrary fuzzy number α on interval [*a, b*],

(11) $$\begin{array}{c} E\left(a\right)=\frac{1}{b-a}\overset{b}{\underset{a}{\int }}E\left(a,x\right)dx \end{array}$$

known as information of *α* at the point, also called information of fuzzy number *α*, and for any fuzzy Number *α* on [*a, b*] has 0 ≤ *E*(*α*) ≤ 1. When *α* is a triangular fuzzy number, the triangular fuzzy number can be calculated from the amount of information according to the definition of triangular fuzzy number and the amount of information:

(12) E(a)=1−2×(u−1)3×(b−a),ρ(a)=E(a)m(a)+(E(a)−1)σ(a).

For the triangular fuzzy number α on the interval [*a, b*], which is called the α' limit coefficient of triangular fuzzy number, where *m*(*α*), *σ*(*α*)  *α* respectively the mean and variance triangular fuzzy numbers. In this method, the limit coefficient considers the fuzzy mean, fuzzy variance, and amount of information influence on fuzzy triangular fuzzy numbers size. Among them, the combination of fuzzy mean value and variance are coefficient directly by the triangle fuzzy number for a given amount of information, the combination of fuzzy mean value and variance coefficient directly by the triangle fuzzy number for a given amount of information, it has high stability, and its calculation process is simple and easy to be programmed, it also has the characteristics of operability.

#### 4.1.2. Enterprise Weight

The basic principle of analytic hierarchy process (AHP) is to give a qualitative description of a set of objectives for paired comparison, by the way of analyzing the relative importance degree of each pair, whereby the quantitative results of each objective weight. Specific methods are as follows.

1. Compare the importance of various customers' needs. This is a process from qualitative to quantitative, through a criterion to judge, comparing the importance of two factors and according to the proportion of 1 to 5 scales to the importance degree of the assignment, the ratio scale as illustrated in Table 2.
2. Forming judgment matrix, the customer requirements fill in the first column of the column and the second column, respectively, which lists the importance degree of each other, as illustrated in Table 3.
3. Using root method to calculate the important weight of customers' needs $\overset{-}{K_{i}^{p}}$, first calculate the absolute important weight *K*_*i*_^*p*^, where *i* = 1,2,…, *n*. For *K*_*i*_^*p*^ standardization $\overset{-}{K_{i}^{p}}=K_{i}^{p}/\sum_{i}^{}K_{i}^{p}$, that is the important weights of customer needs.
4. Verify the validity weight. If C.R < 0.1, it shows that the consistency of a judgment matrix can be accepted; if C.R > 0.1, it shows that data does not need to modify the data consistency, until satisfied, as illustrated in Table 4.

### 4.2. Strategic Weight

(1) The indicators with the quantitative calculation of the first indicators *j* relation *i* option value of the proportion of indicators *p*_*ij*_. Consider

(13) $$\begin{array}{c} p_{ij}=\frac{x_{ij}}{\sum_{i=1}^{m}x_{ij}}. \end{array}$$

(2) Calculation of the first indicators *j* entropy *e*_*j*_ is as follows:

(14) $$\begin{array}{c} e_{j}=-k\overset{m}{\underset{i=1}{\sum }}p_{ij}\ln p_{ij}. \end{array}$$

Among them, *k* > 0. ln is natural logarithm; *e*_*j*_ ≥ 0. If *x*_*ij*_ is all equal to a given *j*, then

(15) $$\begin{array}{c} p_{ij}=\frac{x_{ij}}{\sum_{i=1}^{m}x_{ij}}=\frac{1}{m}. \end{array}$$

*E*_*j*_ gets the maximum at this time; that is,

(16) $$\begin{array}{c} e_{j}=-k\overset{m}{\underset{i=1}{\sum }}\frac{1}{m}\ln\frac{1}{m}=k\ln m. \end{array}$$

If we suppose *k* = 1/ln⁡⁡*m*, there is 0 ≤ *e*_*j*_ ≤ 1.

(3) Calculation of the first *j* indicators of the difference coefficient *g*_*j*_ is as follows. For a given *j*, the smaller the difference of *x*_*ij*_ is, the greater the *e*_*j*_ is; when *x*_*ij*_ are all equal, *e*_*j*_ = *e*_max⁡_ = 1; at this time, there is no effect on indicators *X*_*j*_ for the program compared; the greater the difference between the index values is, the smaller the *e*_*j*_ is, the bigger role of the indicators for the program are compared.

(4) Improving data is as follows. It can be based on expert opinion efficacy coefficient method used for transformation of data and consistency check, taking *X*_*j*_^(*h*)^ = max⁡*X*_*j*_, *X*_*j*_^(*λ*)^ = min⁡*X*_*j*_, the transform using the following formula:

(17) $$\begin{array}{c} X_{ij}^{*}=\frac{x_{ij}-x_{j}^{\left(\lambda \right)}}{x_{j}^{\left(h\right)}-x_{j}^{\left(\lambda \right)}}\times A+B. \end{array}$$

If you consider that the indicator weight should be greater, the date differences in large-scale can be chosen larger and if the data differences are in a small area, it can be chosen smaller. Also, the combination of expert scoring method and the evaluator can add a certain degree of subjective factors, thus increasing the evaluation-oriented, that is, in formula:

(18) $$\begin{array}{c} X_{ij}^{*}=\frac{x_{ij}-x_{j}^{\lambda }}{x_{j}^{\left(h\right)}-x_{j}^{\left(\lambda \right)}}\times \alpha +\left(\alpha -1\right). \end{array}$$

If you want to increase the weights, *α* can be increased, when the data difference is large, and it is large; in a similar manner, if we want to reduce the weight of the indicators, *α* can be decreased, when the data difference is small, and the weight calculated with using the entropy weight method is small.

(5) The definition of weights is as follows. Consider

(19) $$\begin{array}{c} a_{j}=\frac{g_{j}}{\sum_{j=1}^{n}g_{j}}. \end{array}$$

(6) Calculating the value of comprehensive evaluation of *V*_*i*_ is as follows:

(20) $$\begin{array}{c} V_{i}=\overset{n}{\underset{j=1}{\sum }}a_{j}p_{ij}. \end{array}$$

*V*_*i*_ option for the first *i* values of comprehensive evaluation.

## 5. The Numerical Analysis

*(1) The Initial Data of Customer Weight*. See Table 5.

*(2) Enterprise Weight*. See Table 6.

*(3) Strategic Weight*. See Table 7.

*(4) Weight of Functional Objectives on Manufacturing System*.

It is proposed that

(21) $$\begin{array}{c} W^{A}=0.8,\;W^{S}=0.2. \end{array}$$

The comprehensive result is illustrated in Table 8.

It is proposed that

(22) $$\begin{array}{c} W^{A}=0.5,\;W^{S}=0.5. \end{array}$$

The comprehensive result is illustrated in Table 9. Consider

(23) $$\begin{array}{c} W^{A}=0.2,\;W^{S}=0.8. \end{array}$$

The comprehensive result is illustrated in Table 10.

By Table 8, it can be concluded that the six key elements for function objective decision on the manufacturing system in the sequence are personalization, being environment friendly, prompt delivery, low cost, good quality, and high production efficiency. From the reality, the decision results are up to the present market for personalized consumption and premise of legal requirements associated with environmental protection. That could be considered to be personalized and environmental competition orientation; in the overall design, the manufacturing system function objective occupies 57% in total, and the other four for the intensity of competition occupy the proportion of 43%.

From Tables 9 and 10, we can find that this decision method is based on the stability and consistency of weight judgments, if there are remarkable differences on the weight, which need to use additional methods to integrate differences.

## 6. Conclusion

In this paper, we established the three dimensional weight determination methods comprehensively considering the customer needs, enterprise decision-makers intent, and enterprise competitive position. It provides scientific and practical guidance for enterprises in the objective selection of manufacturing system function. From the final calculation of each objective weight, we could see that this method had interaction of human-machine and universal applicability of environment, and it was also easy to operate and compute programming. The deficiency of this paper is that the method still needs a large number of actual inspections, in order to prove it in line with the objective decision of the practical need of the manufacturing system function.

In the future, main job will be to determine the relationship between *W* ^*A*^ and *W* ^*S*^, using the scientific method, such as statistics, data envelopment analysis (DEA) method, and regression method, to determine the proportions of them.

## Figures and Tables

**Table 1:** Linguistic terms and related fuzzy numbers for weight of preference.

Linguistic terms	Fuzzy numbers
(VL)	(0, 0, 0.1)
(L)	(0, 0.1, 0.3)
(ML)	(0.1, 0.3, 0.5)
(M)	(0.3, 0.5, 0.7)
(MH)	(0.5, 0.7, 0.9)
(H)	(0.7, 0.9, 1)
(VH)	(0.9, 1, 1)

**Table 2:** Customer needs relative weight scale.

Scale (*r*_*ij*_)	
1	*i* and *j* are equally important.
2	*i* is slightly more important than *j*.
3	*i* is important than *j*.
4	*i* is more important than *j*.
5	*i* is absolutely more important than *j*.

*r*_*ji*_ = 1/*r*_*ij*_.

**Table 3:** Relative importance for customer needs.

	Efficiency	Cost	Quality	Time	Individuation	Environment
Efficiency						
Cost						
Quality						
Time						
Individuation						
Environment						

**Table 4:** R.I. Value table.

Verify items	1	2	3	4	5	6	7	8	9

R.I.	0	0	0.58	0.90	1.12	1.24	1.32	1.41	1.45

**Table 5:** 

Efficiency	Cost	Quality	Time	Individuation	Environment
(0.3, 0.5, 0.7)	(0.1, 0.3, 0.5)	(0.5, 0.7, 0.9)	(0.3, 0.5, 0.7)	(0.7, 0.9, 1)	(0.9, 1, 1)

Result: (0.13, 0.08, 0.18, 0.13, 0.23, and 0.25).

**Table 6:** 

	Efficiency	Cost	Quality	Time	Individuation	Environment
Efficiency	1	1/2	2	1/3	1/3	1/2
Cost	2	1	3	4	1	2
Quality	1/2	1/3	1	1/2	1/4	1/3
Time	3	1/4	2	1	1/3	1/2
Individuation	3	1	4	3	1	2
Environment	2	1/2	3	2	1/2	1

Result: (0.09, 0.26, 0.06, 0.14, 0.28, and 0.18).

**Table 7:** 

Efficiency	Cost	Quality	Time	Individuation	Environment
0.84	0.79	0.93	0.72	0.96	0.64
0.8	0.72	0.64	0.83	0.9	0.76
0.82	0.68	0.86	0.89	0.78	0.78
0.76	0.8	0.72	0.93	0.9	0.85

Result: (0.13, 0.09, 0.28, 0.18, 0.11, and 0.21).

**Table 8:** The final weight of functional objectives on manufacturing system.

Efficiency	Cost	Quality	Time	Individuation	Environment
0.08	0.12	0.11	0.12	0.32	0.25

**Table 9:** The final weight of functional objectives on manufacturing system.

Efficiency	Cost	Quality	Time	Individuation	Environment
0.11	0.20	0.12	0.27	0.12	0.18

**Table 10:** The final weight of functional objectives on manufacturing system.

Efficiency	Cost	Quality	Time	Individuation	Environment
0.17	0.27	0.18	0.15	0.10	0.13
